# Variability and distribution of spatial evapotranspiration in semi arid Inner Mongolian grasslands from 2002 to 2011

**DOI:** 10.1186/2193-1801-2-547

**Published:** 2013-10-18

**Authors:** David Schaffrath, Christian Bernhofer

**Affiliations:** Institute of Hydrology and Meteorology, Technische Universität Dresden, Dresden, Germany; Professur für Meteorologie, TU Dresden, Fakultät Umweltwissenschaften, PF 1117, 01735 Tharandt, Germany

**Keywords:** BROOK90, MODIS, MAGIM, LAI, Steppe, Xilin, Grazing, Precipitation, Long-term

## Abstract

Grasslands in Inner Mongolia are important for livestock farming while ecosystem functioning and water consumption are dominated by evapotranspiration (ET). In this paper we studied the spatiotemporal distribution and variability of ET and its components in Inner Mongolian grasslands over a period of 10 years, from 2002 to 2011. ET was modelled pixel-wise for more than 3000 1 km^2^ pixels with the physically-based hydrological model BROOK90. The model was parameterised from eddy-covariance measurements and daily input was generated from MODIS leaf area index and surface temperatures. Modelled ET was also compared with the ET provided by the MODIS MOD16 ET data.

The study showed ET to be highly variable in both time and space in Inner Mongolian grasslands. The mean coefficient of variation of 8-day ET in the study area varied between 25% and 40% and was up to 75% for individual pixels indicating a high innerannual variability of ET. Generally, ET equals or exceeds P during the vegetation period, but high precipitation in 2003 clearly exceeded ET in this year indicating a recharge of soil moisture and groundwater. Despite the high interannual and innerannual variations of spatial ET, the study also showed the existence of an intrinsic long-term spatial pattern of ET distribution, which can be explained partly by altitude and longitude (R^2^ = 0.49). In conclusion, the results of this research suggest the development of dynamic and productive rangeland management systems according to the inherent variability of rainfall, productivity and ET in order to restore and protect Inner Mongolian grasslands.

## Introduction

Evapotranspiration (ET) indicates ecosystem functioning in the semi-arid grasslands of Inner Mongolia, since it is controlled by the phenology of vegetation and soil moisture (Zhao et al. 2007 Ketzer et al. 2008) and dominates water balance losses into the lower atmosphere almost entirely in this region (Vetter et al. 2012). Although several field studies on ET exist in the Xilin river catchment of Inner Mongolia, China (Hao et al. 2007,2008 Huang et al. 2010 Miao et al. 2009 and Wang et al. 2012), these studies are restricted by a short period up to a few years and refer to point measurements of a few field sites in this area only. Thus, only little is known about ET of a longer period and about its spatial distribution and variability in this area, where precipitation (P), green vegetation and consequently the components of ET, soil evaporation (E), transpiration (T) and evaporation from intercepted rain (E_i_) are highly variable in both time and space. According to the common variability of these parameters, the grasslands were used by nomads as pastures for centuries. The current livestock management, which is static and exceeds the carrying capacity of the grassland ecosystem, introduced severe problems of degradation and desertification in this area and in Inner Asia in general (Han et al., 2009; Jiang et al., 2006 Brogaard and Zhao 2002 Sneath 1998 Reiche et al. 2009). Tong et al. (2004) reported these grasslands are affected by a decrease of vegetation biomass, height and cover as well as species composition shifts, which slowly convert the typical steppe into a less productive desert steppe. The negative impacts of the ongoing processes of degradation are, e.g., the reduction of the available food resources for the livestock and reduced living conditions for the people due to the increasing vulnerability to erosion. The enormous area of Chinese grasslands (approx. 4 Mio. km^2^) have been a sink area for dust over centuries, but the recent decrease of vegetation, which is mainly caused by overgrazing and unadapted livestock management, prevents dust deposition and promotes wind erosion (Hoffmann et al. 2008 Reiche et al. 2012a). Grassland degradation and its accompanying problems, e.g., dust storms, constitute a central environmental problem for the livelihood of the local population in grasslands and the surrounding Chinese mainland. The results of the intense ecological research activities in these grasslands approve the necessity of the development and adjustment of the land management practices to restore and protect the fragile ecosystems of the Xilin river catchment as suggested by a number of recent publications, e.g., Jiang et al. (2006), Han et al. (2009), Schönbach et al. (2009) and Butterbach-Bahl et al. (2011). The six years of a controlled grazing experiment showed the importance of the seasonal distribution of P, temperature and soil moisture on vegetation dynamics and indicated the complex interaction of grazing, vegetation and climate (Auerswald et al. 2012 Ren et al. 2012). However, these studies and the majority of the ecological grassland studies in Inner Mongolia were conducted in the vicinity of the Inner Mongolia Grassland Ecosystem Research Station (IMGERS) and on the point or plot scale. Regarding the recognised sensitivity of the grassland conditions to the spatial and temporal variability of available water and the dominating role of ET on ecosystem functioning of semi arid regions, the spatiotemporal investigation of ET for a larger area and over a longer period can enhance the understanding of the inherent variability and dynamics of the Inner Mongolian grasslands to support the development of better adapted and sustainable livestock management systems. Hence, the objective of this study is the identification of the spatial and temporal variability of ET and T in the grasslands of the Xilin river catchment over a longer period and the spatial pattern of long-term ET. Therefore, we applied the approach of Schaffrath et al. (2013), which allows for the spatial application of the hydrological model BROOK90 (Federer 2002) and thus, the calculation of ET and its components (E, T and E_i_) during the vast bulk of the rainy season and vegetation period. The approach of Schaffrath et al. (2013) clearly showed the capability of detecting the spatial and temporal distribution of P and ET in this area in 1 km and 8-day resolution with only a few additional ground-based data, mainly needed for model calibration. These data were derived from eddy-covariance (EC) based measurements. The necessary daily meteorological model input and the phenological course of vegetation were derived from 1 km MODIS (Moderate resolution imaging spectroradiometer) leaf area index (LAI) and surface temperature (Ts) data products. The available data enabled to model ET for ten consecutive growing seasons (from 2002 to 2011). Local P was estimated for more than 3000 grid cells in this remote area based on the linear relationship between MODIS LAI gain and measured P around six gauging stations (R^2^ = 0.76, n = 90) (cp. Schaffrath et al. 2013). In this paper we tested the robustness of this approach with a larger dataset (270 observations). In addition, pixel-wise relief information was integrated into the model. Therefore, we derived altitude, slope and aspect (1 km resolution) from the SRTM (Shuttle Radar Topography Mission) data product.

## Material and methods

### Description of the study area

**Figure 1:**
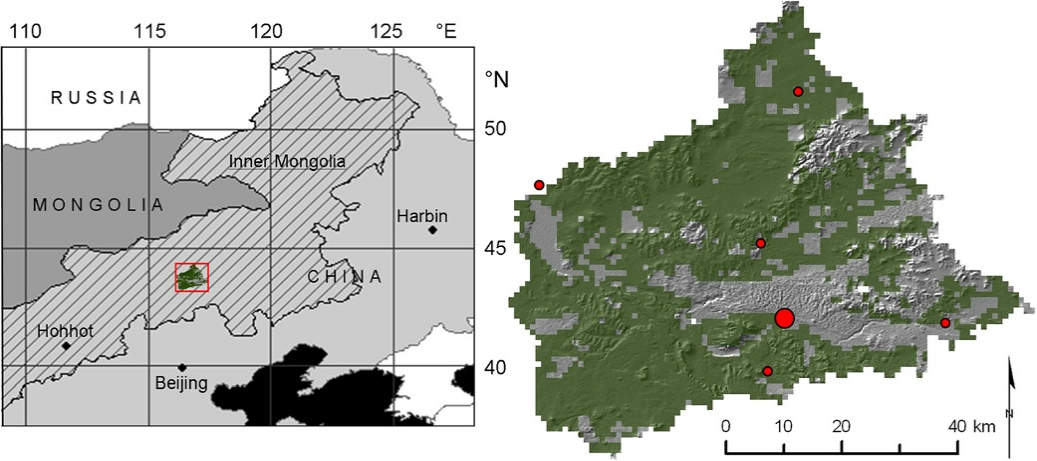
**Location of the study area in Central Asia (left side) and hillshade map (right side) of the Xilin river catchment (grey).** The study area is shown in 1 km resolution and in green colour (grassland or typical steppe), the location of precipitation measurements as red circles, the location of the Inner Mongolia Grassland Ecosystem Research Station (IMGERS) is depicted by the large red circle.

The study area is the typical steppe land use unit (approximately 2600 km^2^) of the Xilin river catchment in the Inner Mongolia autonomous region of China. It is situated in the east of the Mongolian plateau between 44.13° N to 43.41° N and 116.12° E to 117.24° E. The elevation ranges between 1000 and 1500 m above sea level. The relief is characterized by relatively even plains, gentle hills, mountains and high plateaus. Figure 1 shows the location and a hillshade representation of the study area.

The typical steppe covers 72% or 2600 km^2^ of the catchment and is considered representative of semi-arid temperate grasslands which are stretched over large parts (more than 40%) of China (Akiyama and Kawamura 2007 Fan et al. 2007 Kawamura et al. 2003). The Institute of Botany of the Chinese Academy of Science maintains the Inner Mongolia Grassland Ecosystem Research Station (IMGERS) in this area. IMGERS was established in 1979 to study the drivers and the functioning of the grassland ecosystem and focuses on the development of sustainable ecosystem management as well (Kang et al. 2007). These grasslands are comparably extensively researched, including the studies of the interdisciplinary Sino-German Research Group MAGIM (Matter fluxes in Grassland Ecosystems of Inner Mongolia), which were also conducted at IMGERS and its experimental field sites since 2004 (cp. Butterbach-Bahl et al. 2011).

In the study area, the climatic conditions are characterised by a strong contrast of long, cold and dry winters and short, warm and relatively wet summers. Spring and autumn are short transitional seasons (Domroes and Peng 1988). While the mean annual temperature is 1°C, the January mean temperature is −20°C and in July it is 21°C (Kawamura et al. 2005); the frost-free period generally lasts between 80 and 100 days. The amount and distribution of precipitation is highly variable and over 70% of the annual P falls between May and August (Chen et al. 2005), when moist air masses are transported from South-China and Japan towards this region. The long term annual mean P at IMGERS (1982–2005) is 330 mm, however annual P varies between 170 and 500 mm. Tong et al. (2004) and Schaffrath et al. (2011) reported the decline of P in northern directions in the Xilin river catchment.

According to the World Reference Base for Soil Resources (WRB), the most common soil groups of the study area are *Phaeozem*, *Chernozem* and *Kastanozem* which differ in colour, thickness and the amount and depth of secondary carbonates but they are also characterised by relatively small differences of soil texture, since sand is the dominant particle fraction (cp. Wiesmeier et al. 2011 Barthold et al. 2013). The vegetation of the study area is characterised by C3 grasses, mainly *Leymus chinensis* and *Stipa grandis*. However, degraded areas are characterised by a decrease of these species and an increased appearance of *Cleistogenes squarrosa* and *Artemisia frigida* (Tong et al. 2004). As these grasslands are the primary natural resources in this area and as they are referred to as the pastures of highest quality in China (Chen et al. 2008), they are almost entirely used for livestock grazing (Wiesmeier et al. 2011).

### The hydrological model BROOK90

For this study, we used BROOK90 (Federer 2002 Federer et al. 2003), a sophisticated and process-oriented hydrological model that simulates the components of the total ET, transpiration (T), evaporation from intercepted rain (E_i_) and soil evaporation (E), by a physically-based two-layer model (Shuttleworth and Wallace 1985), as well as vertical soil water movement and streamflow for a certain location at a daily time-step. BROOK90 has been applied for a variety of water balance related studies, e.g., Combalicer et al. (2010), Armbruster et al. (2004), Schwärzel et al. (2007,2009), Vilhar et al. (2010) and Schaffrath et al. (2013). The model is parameter-rich: soil hydraulic parameters and root density are parameterised for several layers below ground, since matrix potential and hydraulic conductivity are essential for the simulation of soil water movement. Here, BROOK90 uses a modification of the Campbell (1974) expression with a near-saturation interpolation of the Clapp and Hornberger (1978) formulation. In BROOK90, canopy characteristics are described by year-round specification of LAI and vegetation height changes over the year. ET is modelled by a Shuttleworth and Wallace (1985) approach, which in turn is based on the Penman-Monteith equation (PM) here shown for latent heat flux (*L.ET*), Eq. (1):
1

where *L.ET* is the latent heat flux from the canopy (W m^-2^), *Δ* is the rate of change of saturation vapour pressure with temperature (Pa K^-1^), *R*_n_ is the net radiation above the surface (W m^-2^), *G* is the ground heat flux (W m^-2^), *c*_p_ is the specific heat capacity of air at constant pressure (J kg^-1^ K^-1^), *ρ*_a_ is the density of air (kg m^-3^), *VPD* is the vapour pressure deficit in the air (Pa), *γ* is the psychrometric constant (Pa K^-1^), *r*_c_ is the canopy resistance (s m^-1^), and *r*_a_ is the aerodynamic resistance between the canopy and a reference height at which *VPD* is measured (s m^-1^).

T and E are calculated separately by the Shuttleworth and Wallace (1985) modification of the PM equation where:
2

and:
34

with *VPD*_0_ the vapour pressure deficit at the effective source height (Pa), *A* is *R*_n_ - *G* or the available energy above the canopy (W m^-2^), and *A*_s_ is *R*_ns_ - G or the available energy at the ground (W m^-2^), *R*_ns_ is the net radiation above the soil surface (W m^-2^). The different water vapour and sensible heat pathways and resistances from the soil and from the canopy are taken into account by the model, since resistances are defined separately as: canopy surface resistance (*r*_sc_), resistance to restrict vapour movement from the leaf surfaces to the effective source height for water vapour in the canopy (*r*_ac_), resistance to movement of water vapour from inside the soil to the soil surface (*r*_ss_) and resistance to vapour movement from the soil surface to the source height (*r*_as_).

In general, potential T is based on maximum leaf conductance and vapour pressure deficit (VPD). However, actual T is reduced below potential T when water supply to the plant is limited by plant resistance, rhizosphere resistance and minimum leaf water potential (Federer et al. 2003). The aerodynamic resistance varies according to leaf area index and canopy height; it is modified from Shuttleworth and Gurney (1990). E is calculated from soil water potential in the top soil layer; potential E_i_ is calculated indirectly from the existing soil surface wetness of the Shuttleworth and Wallace equation and the canopy height (Federer 1996). Further and more detailed information about BROOK90 are provided by Federer et al. (2003); a helpful and detailed tool is the online documentation of the model (Federer 2002).

### Parameterisation of BROOK90

Micrometeorological data of the MAGIM project were used for the initial parameterisation of BROOK90 (Vetter et al. 2012): between 2004 to 2009 eddy covariance (EC) measurements were conducted at several grassland sites differing in livestock management (ungrazed, winter grazed, continually grazed, heavily grazed) to obtain representative data on the conditions of typical steppe in the Xilin river catchment (Ketzer et al. 2008 Wang et al. 2012). The EC towers were equipped with three-dimensional sonic anemometers *CSAT3* (Campell Scientific, USA) to detect the turbulent fluctuations of temperature and wind. *LI-7500* (LI-COR Inc., USA) infrared gas analysers were used for the measurement of H_2_O and CO_2_ concentrations. Ketzer et al. (2008) and Wang et al. (2012) describe the setup design of these towers in more detail. The measured and calculated values of ET, radiation flux density, soil heat flux density, air temperature, humidity, and precipitation as well as information on LAI and soil parameters were used for the initial parameterisation of BROOK90, which concentrated on the period from May through September since there is little to no plant activity from October to April. Also, ET is very small during this period. Since the model does not allow for year-round specifications of albedo, it was set to 0.2 as suggested for temperate grasslands by Federer et al. (2003). This value is also in good agreement to our measurements and research (Fan et al. 2007 Ketzer et al. 2008). Canopy conductance (g_c_), which determines transpiration and thus, largely influences the simulated output, was estimated from daily ET (converted to latent heat flux *L.ET*) of the EC measurements using a re-arranged PM equation (Eq. 5):
5

where: *r*_c_ is the canopy resistance (s m^-1^), *β* is the Bowen ratio (H L.ET^-1^), *r*_*a*_ is the aerodynamic resistance (s m^-1^) which was estimated according to Thom and Oliver (1977), *ρ*_*a*_ is the density of the air (kg m^−3^), *c*_*p*_ is the specific heat capacity of air at constant pressure (J kg^−1^ K^−1^), *γ* is the psychrometric constant (Pa K^-1^) *VPD* is the saturation vapour pressure deficit (Pa), and *L.ET* is the latent heat flux (W m^-2^).

According to the analysis, g_c_ showed a clear dependency on VPD and soil moisture and the typical maximum g_c, max_ (95% quantile) was 6 mm s^-1^. The model uses maximum g_c, max_ according to Jarvis (1976); depending on the effects of temperature, VPD, soil moisture and incoming short-wave radiation on stomatal opening, g_c_ is set to a fraction of g_c, max_.

**Figure 2:**
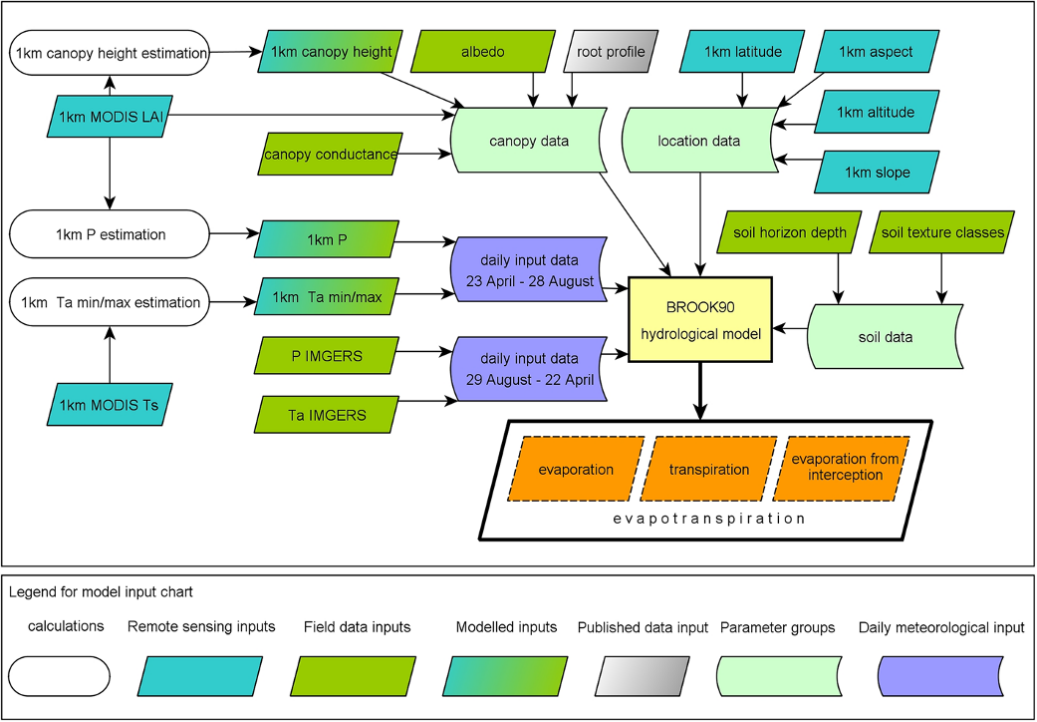
**Flowchart of the sources and organisation of model input and parameters for the calculation of spatial ET.**

A more detailed background on the initial parameterisation of BROOK90 for the grasslands of the study area, validation and simulated results at the experimental field sites of MAGIM are summarised by Vetter et al. (2012) and Schaffrath et al. (2013). This study is based on the initial parameterisation of the model with several modifications for the application in a much larger area. An overview of the organisation of parameters and daily input data are shown in Figure 2. The concept is based on the work of Schaffrath et al. (2013) who calculated ET separately for every 1 km^2^ pixel of the study area. Individual parameters were used if available (e.g. LAI, latitude, slope and aspect), but some general assumptions were necessary due to limited field data: the same relative root distribution over depth has been used as described for temperate grasslands according to Jackson et al. (1996) and Federer et al. (2003) and one standard soil profile for all pixels was generated, although the analysis of soil texture of more than 30 soil profiles sampled within the study area by the MAGIM project (Wiesmeier et al. 2009 Wiesmeier et al. 2011 Steffens et al. 2011) showed some variations in the partitioning of the material. However, sand is the dominating particle fraction in the soils of the study area and the effect of the variations in texture on ET was found to be relatively small: the entire range of observed texture profiles was simulated to quantify the effect of the variations on ET in a small test area in Vetter et al. (2012). Then, three soil profiles that resulted in the minimum, mean and maximum ET were simulated for the whole study area in 2006 by Schaffrath et al. (2013). The results showed some effects on ET which deviated between −11% and +6% from ET simulated with the soil profile used in this study that is considered representative for the study area as it is resulting in average values of ET (Table 1). Also, since BROOK90 deduces soil water retention and movement from soil texture classes, minor differences are neglected by the model. Individual LAI was taken from the MODIS MOD15 data product (collection 5) and the dates and values of the most significant LAI changes were parameterised during the course of every year. MOD15 LAI is available every 8 days and contains the highest LAI at a pixel during an 8-day observation period. BROOK90 provides 10 supporting points for the annual LAI course. The LAI of DOY (Day of the year) 1 and DOY 366 were set to zero for all pixels. DOY 113 (23 April) and DOY 240 (28 August), which are the start and the end dates of the study period, were linked with the LAI at that time. The remaining six dates were filled with the DOY and LAI of the six most significant LAI-changes. Vegetation height (h, in cm) was roughly estimated (h = 35.5 LAI, R^2^ = 0.49), based on a relationship between measured vegetation height and LAI data from MAGIM as well as from publications (Fan et al. 2009 Zhang and Zhao 2009). These data were implemented analogously to LAI. Information on latitude was taken from the MODIS coordinates. Individual 1 km values of altitude, aspect, and slope were calculated from the SRTM (Shuttle Radar Topography Mission) data product.

**Table 1:** **Parameters of the soil profile used in this study**

Soil horizon	Texture class	Thickness (cm)	S%	ψ _f_ kPa	θ_f_m^3^ m^-3^	θ_s_m^3^ m^-3^	K _f_ mm d ^-1^
A1	sandy clay loam	45	0	−6.3	0.317	0.420	4.2
A2	sandy loam	23	0	−7.9	0.266	0.435	5.5
C	loamy sand	50	0	−3.8	0.203	0.410	3.5

Soil hydraulic parameters and thickness were deduced from texture of available data (> 30 samples of soils in the typical steppe land use unit of the Xilin river catchment) after Clapp and Hornberger (1978), provided by Federer (2002), the most important soil parameter input for BROOK90 are stone fraction (S), matrix potential at field capacity (ψ_f,_), volumetric water content at field capacity (θ_f_), porosity / water content at saturation (θ_s_), hydraulic conductivity at field capacity (K_f_).

### Calculation of daily model input

One important reason for using BROOK90 in this study is the ability of the model to derive necessary calculation input from a few daily meteorological input data once the model is parameterised. The minimum daily input data set consists of precipitation (P), minimum air temperature (Ta_min) and maximum air temperature (Ta_max) to determine daily ET. Additional information on solar radiation, wind speed and vapour pressure are desirable, but not essential, since they are also generated by BROOK90: solar radiation is estimated from known information of latitude, slope, aspect and DOY by multiplication with a constant (0.55) for typical atmospheric attenuation; wind speed at 10 m is set to constant 3 m s^-1^ and vapour pressure is estimated from saturation vapour pressure at Ta_min (Federer 2002). The use of the approximated values from 8-day Ta data results in the loss of effects in daily variation, but these effects are assumed to be small because comparison of the ET simulated with the full measured data set were compared with ET simulated with input data generated by BROOK90 by Vetter et al. (2012), who showed relevant effects to be well described and general trends in ET not to be affected. Also, the calculated ET results are presented with a temporal resolution of 8 days.

### Estimation of spatial precipitation

**Figure 3:**
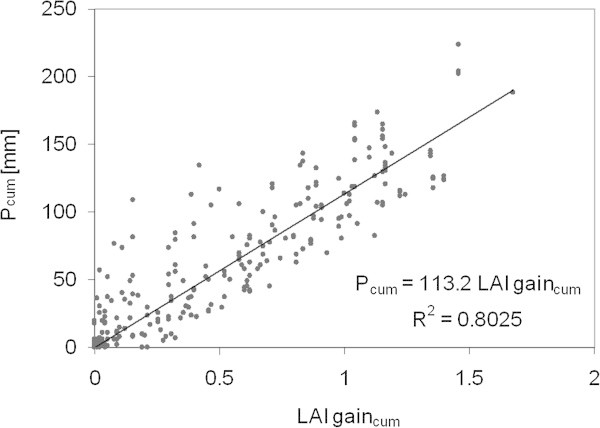
**The relationship between the measured cumulative P from six stations and the corresponding mean cumulative gain of MODIS LAI (n = 270).**

P was modelled for every grid cell on the basis of a linear relationship between measured P sums and the LAI gain of the MODIS MOD15 LAI data product (collection 5) of a 3x3 pixel domain around the P measurements (Figure 1 and 3). The LAI gain is defined as the positive difference of two corresponding pixels in two consecutive MODIS LAI captures (8-day interval) in which the LAI observed at the later date is used as the minuend.

The empirical model was firstly described by Schaffrath et al. (2013). Here, we present an improved version with more observations (n = 270) during three years of measurements (2005, 2006 and 2008) resulting in a more robust relationship (R^2^ = 0.80) compared to the old version with data of 2006 only (R^2^ = 0.76, n = 90). In general, the model (cumulative LAI gain vs. P sums of all measurements) is translating into a mean of 11.3 mm of P for a LAI gain of 0.1 and vice versa. Among the six stations, site specific relationships exist, ranging from 9.5 mm to 14 mm. The old version of the model (Schaffrath et al. 2013) showed a larger uncertainty due to the wider range of site specific relationships (from 10 mm to 21 mm). However, P for each grid cell was calculated using the mean relationship for every 8-day interval, Eq. 6:
6

Eq. 6 was applied to the calculated LAI gain of a 3x3 averaging kernel for each pixel. This method was applied until the end of August (28 August, DOY 240) only; Schaffrath et al. (2013) revealed problems in detecting reliable P beyond this date, because LAI in general no longer increases due to reduced radiation and temperature triggering the begin of senescence.

The thus derived 8-day P values were scaled to daily values with a cascade model after Olsson (1998). This step was required because BROOK90 works with daily input of P. The scaling cascade model was trained with P time series from all available stations (60 years in total). In a next step, the disaggregated P was corrected for losses due to wind, according to Richter (1995), Eq. 7:
7

where *b* and *ϵ* are coefficients that describe station parameters and the type of P. According to Richter (1995) *b* was set to 0.345 and *ϵ* to 0.38 correcting P for a free-standing station in flat terrain measuring rain between April and September.

### Estimation of spatial air temperatures

For the calculation of ET with BROOK90, daily input of minimum and maximum air temperatures are obligatory. They were generated from the MODIS 8-day Ts (surface temperature) product MOD11A2. The product provides day-time and night-time temperatures and Vetter et al. (2012) found a well-established relationship between the measured minimum air temperatures (*Ta* _ min _*m*_) at our stations and the Ts calculated by MODIS aboard the TERRA satellite at night (*Ts_TN*), which was used to derive pixel-wise minimum air temperatures (*Ta* _ min _*mo*_) for this study: over the period from 2005 to2008 (23 April to 30 September), the 8-day mean *Ta* _ min _*m*_ and *Ts_TN* showed a high correlation (R^2^ = 0.92, n = 80) which allowed for the calculation of minimum air temperatures from the relationship shown in Eq. 9:
8

The Ts of the MOD11A2 data product was also used to model maximum air temperature (*Ta* _ max _*mo*_): Vetter et al. (2012) showed it correlates relatively well (R^2^ = 0.88, n = 80) with the measured maximum air temperature (*Ta* _ max _*m*_) at the stations, when calculated after Eq. 9:
9

where *Ts_TD* is the TERRA MODIS Ts at daytime. Since *Ta* _ min _*mo*_ and *Ta* _ max _*mo*_ were derived from the MOD11A2 data product, that provides the average temperatures during 8-day intervals, *Ta* − min _*mo*_ and *Ta* _ max _*mo*_ were treated as daily values changing every 8 days in BROOK90.

### Generation of daily model input from 29 August to 22 April

Model input outside the study period (from 29 August to 22 April) was not derived from MODIS data, because the method of estimating P from LAI cannot be applied during this time and the MODIS Ts product may shows an increased number of missing values due to snow cover in the region. Therefore, the daily input during this period was provided from other reliable sources: from 2001 (the 2001 model run was used to initiate the model) to 2009, meteorological data of IMGERS was used. Data gaps were filled with data of our micrometeorological measurements. Unfortunately, IMGERS data were not available to us in 2010 and 2011 and our stations only collected data until 2009. Thus, daily Ta was calculated from the mean Ta of IMGERS between 2001 and 2009; daily P was filled from P at IMGERS in 2002 (average P during this period). We are aware of the loss of the spatial variation in the daily model input, but we assume little influence on the modelled results, because air temperatures are generally below 0°C and P was very low (mean value = 53 mm during this period) as well.

### Multi-year spatial application of BROOK90

BROOK90 version 4.4e software was used with an adaptation for batch processing. Every pixel in this study was calculated and treated separately. There is no interaction between adjacent pixels. The model simulated ET for every pixel with its individual location parameter set, canopy parameters (LAI and canopy height changes over the year) and daily input data set (P, minimum and maximum air temperature). The simulations started with an initial run (2001 data) to generate initial values of snow storage, groundwater storage and matrix potential of each soil layer for 2002. Analogously, the final values of every year were transferred to the start of the following year.

### Comparison with MODIS evapotranspiration

A global 8-day 1 km spatial ET data product (MOD16) is generated by the MODIS science team. It is available online (http://www.ntsg.umt.edu/project/mod16, accessed 20 January 2013). The calculation is also based on the PM equation and the datasets are estimated using the ET algorithm by Mu et al. (2007 and 2011) that is based on the work of Cleugh et al. (2007), who estimated 16-day evapotranspiration for Australia using MODIS data and surface meteorology. A comparison of our results with the MODIS data product is highly useful, not for strict validation of either ET, but it is a possibility to check their plausibility and to identify similarities and differences in both data sets. Also, based on the low number of published works, we assume the scientific community seems to hesitate using the globally available MOD16 ET product despite the need of spatial information on ET for a broad range of applications. Although the MOD16 ET does not separate E and T, it is an exclusive opportunity to compare our ET results with spatial ET that is already available in the same spatial and temporal resolution and over the same period (2002–2011).

## Results

### Spatiotemporal variability of evapotranspiration

**Figure 4:**
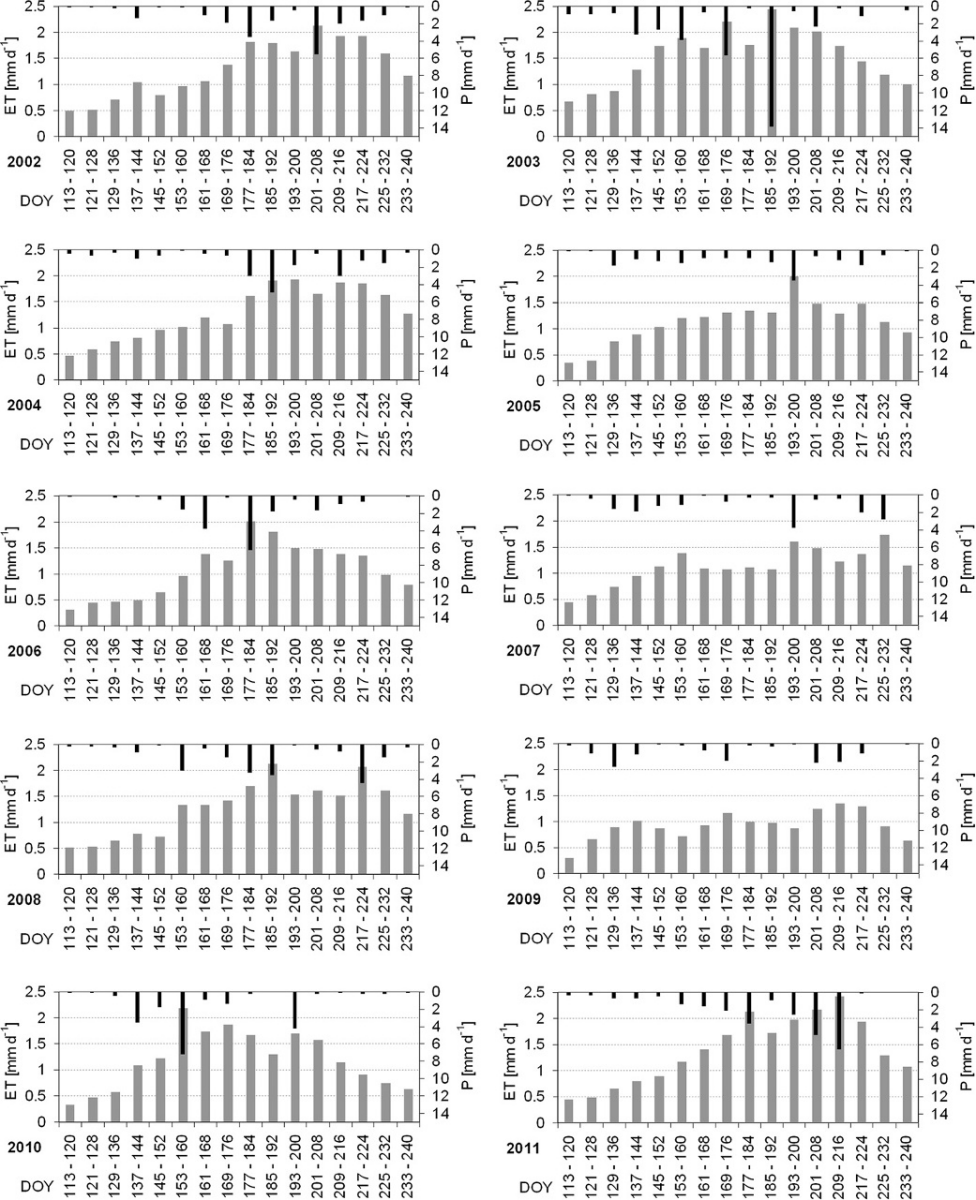
**The annual courses (2002 to 2011) of the mean ET (grey bars) and P (black bars) in the study area from 23 April (DOY 113) to 28 August (DOY 240).**

The characteristics of the annual courses of the mean ET in the study area are quite diverse (Figure 4), but ET is low (between 0.3 mm d^-1^ and 0.6 mm d^-1^) at the end of April (DOY 113–120) in every year. A substantial increase of ET occurs in every year, but with significant differences in the date and in the magnitude of the increase: in some years, spring and early summer ET increased gradually (e.g., in 2004, 2007 and 2011). In 2006 and 2008 a considerable increase of ET occurred late, at the beginning of June (between DOY 153 and DOY 160). The mean maximum ET of 8-day intervals in the grasslands ranged from very low 1.4 mm d^-1^ in 2009 up to 2.4 mm d^-1^ (in 2003 and 2011). However, the highest ET of a single pixel was 5.2 mm d^-1^ during an 8-day period in July of 2003 (DOY 185–192). The date of peak-ET was highly variable too; it occurred very early in 2010 (at the beginning of June, DOY 153–160) and very late in 2007 (in the mid of August, DOY 225–232). Also, the temporal distribution of ET showed a unimodal distribution in 2005 and 2006, a bimodal distribution in 2008 and a multimodal distribution, e.g., in 2002 and 2004. In general, ET declines in August and is between 0.6 mm d^-1^ and 1.3 mm d^-1^ during the last 8-day interval of the study period (DOY 233–240).

The mean ET sum of the study area and annual study period (23 April to 28 August) was 158 mm from 2002 to 2011 with a standard deviation (SD) of 22. mm and a coefficient of variation (CV) of 14%. Lowest ET was modelled for 2009 (119 mm) and highest ET for 2003 (199 mm). In general, annual ET equals annual P (ET/P = 0.99), however in 2003 when P was much higher (301 mm) than the average annual P of our study period (165 mm), the linear relationship between annual P and ET is not valid, since ET was significantly lower (199 mm) than P (Table 2). The analysis of the 2003 data revealed simulated vertical flow below the soil profile of 76% (77 mm) of the difference between P and ET. The remains (25 mm) were stored in the parameterised (1.2 m) soil profile; in all the other much dryer years the moisture stored in the soil decreased between 23 April and 28 August as Table 2 shows.

**Table 2:** **Overview of the summed-up model results from 23 April to 28 August**

	2002	2003	2004	2005	2006	2007	2008	2009	2010	2011	MEAN
P [mm]	168.2	300.8	160.9	141.7	136.3	141.4	162.1	116.4	150.5	174.6	165.29
ET [mm]	167.5	198.6	165.4	145.5	139.6	145.8	165.1	119.2	154.5	179.7	158.09
T [mm]	89.7	113.0	92.7	83.3	80.2	79.2	88.1	69.4	91.1	102.4	88.91
E [mm]	66.8	73.5	61.3	54.4	50.5	59.1	66.0	44.7	56.5	63.6	59.64
Ei [mm]	11.0	12.1	11.4	7.8	8.9	7.5	11.0	5.1	6.9	13.7	9.54
VRFL [mm]	13.7	77.3	10.9	3.8	4.2	3.5	8.8	1.5	14.1	18.6	15.64
P-ET-VRFL [mm]	−13.0	24.90	−15.40	−7.60	−7.50	−7.90	−11.80	−4.30	−18.10	−23.70	−8.44
ET/P	0.99	0.66	1.03	1.03	1.02	1.03	1.02	1.02	1.03	1.03	0.99
T/ET	0.54	0.57	0.56	0.57	0.57	0.54	0.53	0.58	0.59	0.57	0.56

Note VRFL is the vertical flow through the parameterised soil and P-ET-VRFL corresponds to the change in soil moisture during that time.

**Figure 5:**
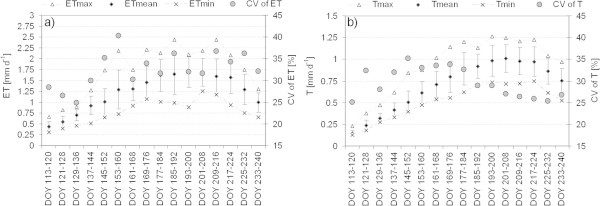
**Temporal variability of a) ET and b) T in the study area from 2002 to 2011.** The figure shows minimum, mean and maximum values of ET and T of the study area as well as their standard deviations and the mean coefficient of variation (CV) of spatial ET.

The band between minimum and maximum ET in Figure 5a shows the spectrum of the magnitude of ET in the study area from 2002 to 2011 and the CV indicates the mean interannual ET-variability in the study area in 8-day intervals. The 8-day CV of ET is generally high and variable and significantly higher than the CV of annual ET in the study area. Figure 5a) shows a substantial increase of the variability from minimum (25%) around mid of May (DOY 129–136) to maximum (40%) at the begin of June (DOY 145–160) indicating an increase of uncertainty of ET in the study area at that time.

**Figure 6:**
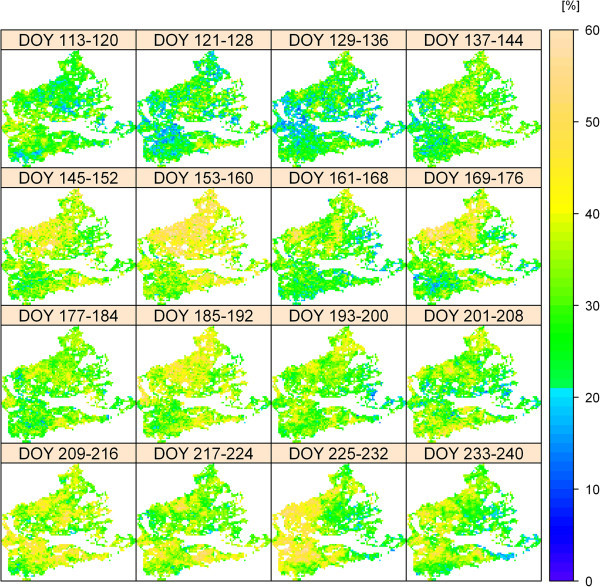
**Illustration of the spatiotemporal variability of ET in 8-day intervals in the study area.** The coefficient of variation (CV) was used to quantify the variability of ET from 2002 to 2011; the higher the CV, the higher the variability. For reasons of visibility, the figure does show the CV up to 60%, though the CV of a few pixels was higher (up to 75%).

**Figure 7:**
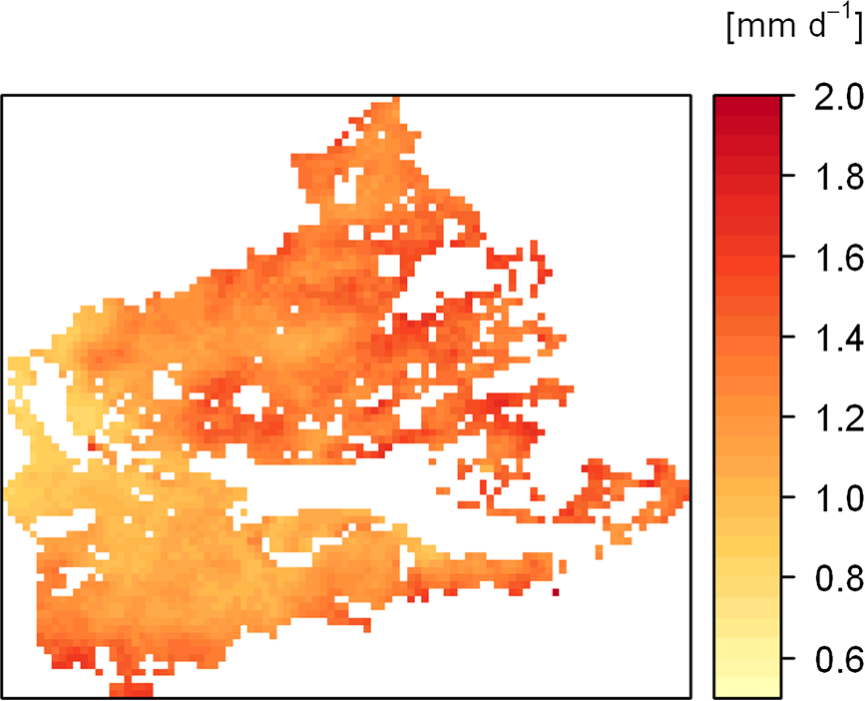
**The spatial pattern of the long term (2002–2011) mean spatial distribution of ET in the grasslands of the Xilin river catchment.**

The spatiotemporal CV of ET from 2002 to 2011 is shown in Figure 6. In contrast to the mean values for the whole area of Figure 5a, the individual pixels in Figure 6 show a wider spectrum of variability with a CV of up to 75%. Also, the spatial pattern and amount of the CV is highly variable over the course of the year. Despite high interannual and innerannual variations of spatial ET, the ten years of modelled ET indicate the existence of a long term spatial pattern of ET with areas of very low mean ET (0.77 mm d^-1^) and areas with more than twofold ET (1.99 mm d^-1^). In general, ET is higher in the eastern part of the study area (Figure 7) and the mean ET for the grasslands over the whole period is 1.24 mm d^-1^

### The partitioning of ET into E and T and their variability

The annual courses and the amount of E and T as well as their ratio are very variable, too. E and T are very low (below 0.5 mm d^-1^) at the beginning of the study period (end of April). In general, E is slightly higher than T until the end of May. In some years, e.g., 2002 and 2004, a substantial increase of E or T does not occur until the beginning of June (DOY 153–160); in other years, e.g. 2003 and 2010, the increase of both E and T was earlier, in mid May. Although the amount and course of E and T are quite variable, E generally dominates over T in spring. T will increase and generally dominates over declining E in summer. However, in spring of 2006, T dominated over E and two distinctive peaks of E occurred around mid and end of June. Also, large differences of the amount of E or T are quite common between two consecutive 8-day intervals. At the end of each years study period (end of August), E was very low again (below 0.5 mm d^-1^) and T varied between 0.5 mm d^-1^ (2009 and 2010) and 1 mm d^-1^ (2002 and 2004). The variability of 8-day T observed in the study area during the 10 years is shown in Figure 5b. Although the CV of T varies between 25% and 35% and thus, is lower than the CV of ET (cp. Figure 5a), it also increases in spring and early summer. However, in July the CV of T drops below 30% and is further decreasing. The widening band between the mean minimum and maximum T shows a broad spectrum of modelled T in the grasslands throughout the analysed vegetation periods.

**Figure 8:**
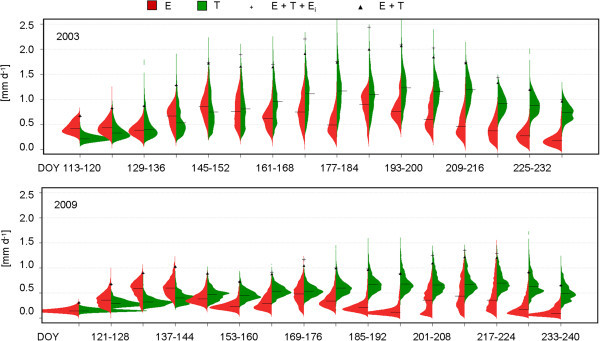
**Probability density functions and median (black horizontal line) of the spatial distribution of the components of ET in two selected years (2003 and 2009).** Evaporation is depicted on the left side in red and transpiration is shown on the right side in green. The + shows ET as the sum of its three components and the triangle shows ET without E from intercepted rain.

**Figure 9:**
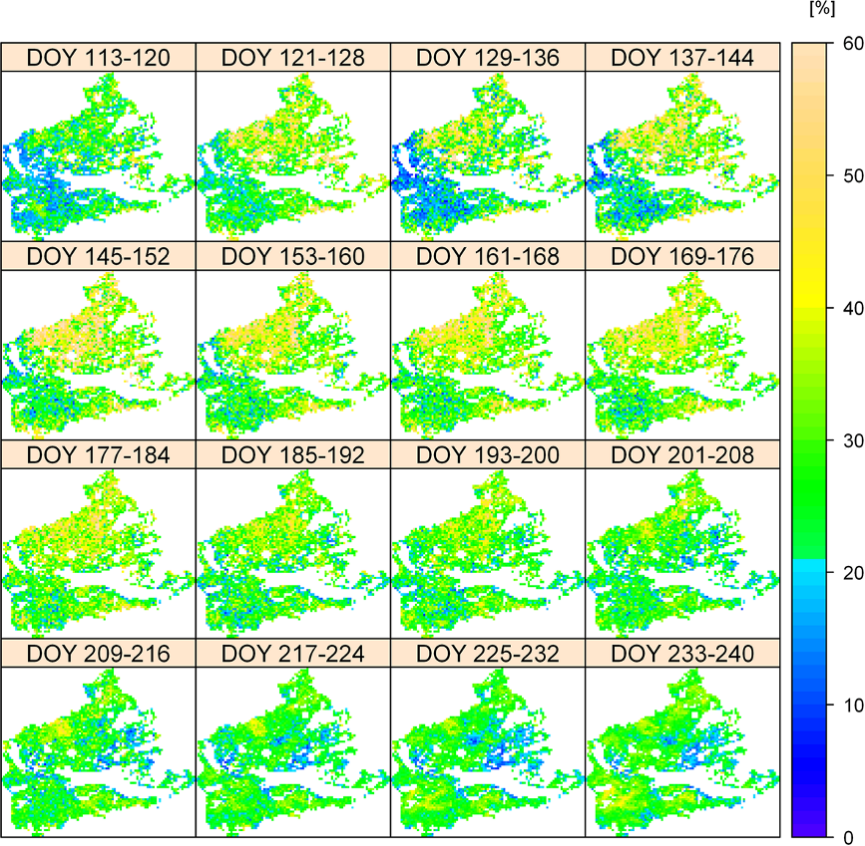
**Spatiotemporal variability of T in 8-day intervals in the study area from 2002 to 2011.**

The differentiated courses of E and T are shown as the probability density functions (PDF) and the courses of the components of ET (E, T and E_i_) for a very wet (2003) and very dry year (2009) in Figure 8: here, the left side in the beanplots shows the PDF of all pixels for E (red colour) and the right side shows the PDF of T (green colour). While E and T as well as their spatial differences are small during the first three 8-day intervals (DOY 113–136) in both years, the stretched PDFs of E and T show a substantial increase and also the more pronounced spatial differences in 2003 afterwards. A slight increase of T occurs in 2009, but compared to the 2003 data, E and T stay on a very low level throughout the whole study period. E_i_, which is visible from the difference between the + and the ▲in the figure, is also very small in 2009. In 2003 there are several 8-day intervals (e.g., from DOY 169–176 and DOY 185–192) with considerable amounts of E_i_, up to 0.4 mm d^-1^ or approx. 15% of ET indicating high LAI and P. The spatiotemporal distribution of the variability of T is displayed in Figure 9. Here, the calculated CV of T is shown analogously to Figure 6 every 8 days, from 2002 to 2011. However, the spatial distribution of the CV of T is distinct from the spatial variability of ET in Figure 6. The spatial patterns of the CV in Figure 9 are different for each 8-day interval, but there are similarities, e.g., the CV is lower in the western and south-western part of the study area in spring and early summer (until approx. DOY 184) than the CV in the northern part. Afterwards, the difference between these areas is less pronounced, e.g., due to a decrease of the CV of T in the northern areas. Table 2 shows the T, E and E_i_ sums (23 April-28 August) as well as the T/ET ratios from 2002 to 2011. T was higher than E throughout the study period and the ratio between T and ET was relatively balanced (between 0.53 and 0.59) regardless of the amount of P. E_i_ accounts for 4% to 8% of ET.

### Comparison of BROOK90 evapotranspiration with MOD16 evapotranspiration data

**Figure 10:**
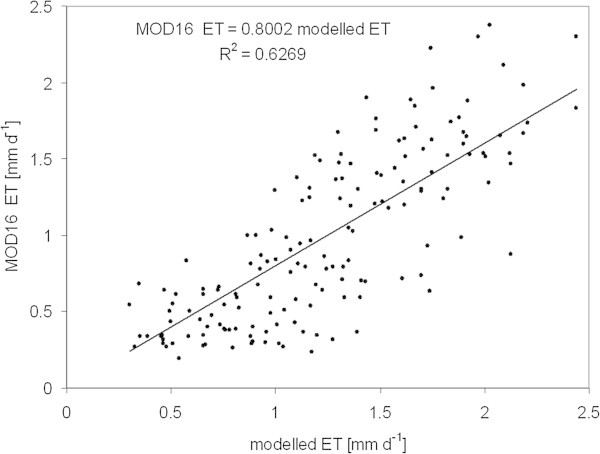
**The relationship between MODIS MOD16 ET data and modelled BROOK90 ET.** Regression is based on mean values of 8-day intervals in the study area (2002–2011).

In general, the spatial mean values of ET of the MOD16 product are in the range of our modelled ET in the study area. Both simulations of ET show similarities in the annual courses of ET, but ET of the MOD16 data product is always lower in spring and early summer than the ET modelled by BROOK90. Figure 10 shows the spatial mean values of ET in the study area of both data sets (8-day values). According to the regression, MOD16 ET accounts for about 80% of our modelled ET (R^2^ = 0.63, n = 160), mainly due to the lower ET values in spring.

**Figure 11:**
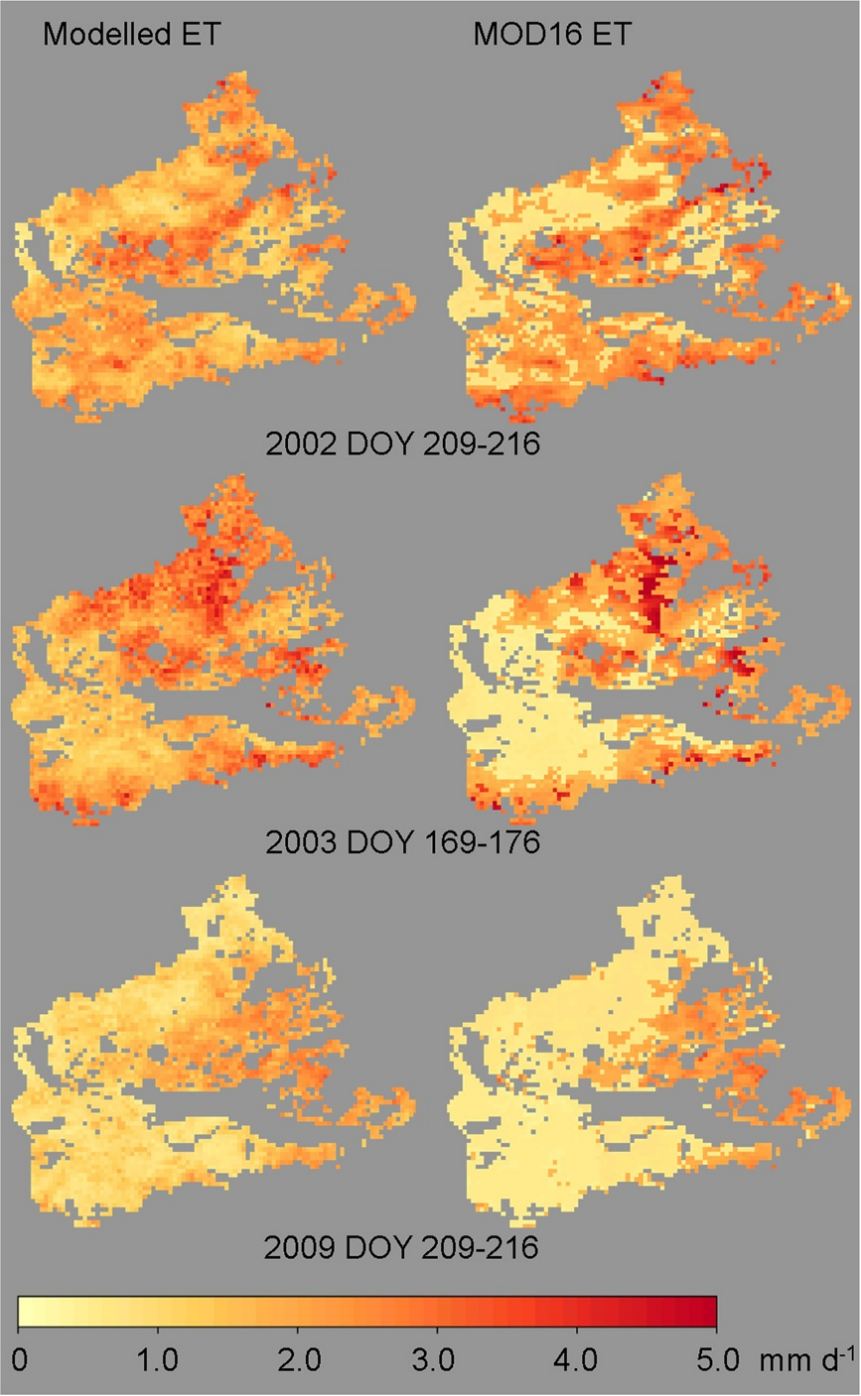
**Examples of the spatial distribution of the comparison between modelled BROOK90 ET and MODIS MOD16 ET.**

However, in summer, MODIS ET sometimes exceeds our modelled ET. The correlation of annual ET is slightly higher (R^2^ = 0.79, n = 10) and on the annual basis MOD16 ET is also 80% of ET modelled by our method. Some differences exist between both data sets when comparing the spatial ET, but in most scenes the spatial pattern is similarly (Figure 11). However, the MOD16 ET in general has a higher contrast than our modelled results, which show a finer spatial differentiation of ET.

The long-term spatial pattern of MOD16 ET (over the whole study period from 2002 to 2011) also indicates low mean ET in the western parts of the study area. Linear regression of the spatial patterns of both data sets (MOD16 and BROOK90, n = 3029) resulted in R^2^ = 0.83 or in R^2^ = 0.77 when forced through the origin. The summary statistics of the mean spatial ET confirmed the lower ET of the MOD16 data product (mean value = 0.97 mm d^-1^) and the slightly higher contrast (range from 0.54 to 1.83 mm d^-1^) when compared to the spatial pattern of ET modelled with BROOK90 (mean value = 1.24 mm d^-1^, range from 0.77 to 1.99 mm d^-1^).

## Discussion

### Modelled results

For the first time, a long-term study of spatial ET was conducted for the grasslands of Inner Mongolia. This is of particular importance, as ET can not only be considered a key component of the water cycle in this region or regarded as water flux into the atmosphere, but ET also integrates other environmental factors, e.g. the abundance of P, and its interactions with soils and vegetation. The analysis of the model results revealed a pronounced spatial and temporal variability of innerannual and interannual ET, in which the variations of ET were mainly governed by the distribution of P. The temporal (8-day) distribution of P was quite diverse and the annual courses of ET and the high CV of ET reflect this variability very well. The date and amount of peak-ET of the whole study area were also very variable: in this study peak-ET was reached between the beginning of June and mid August and it varied between 1.4 mm d^-1^ and 2.4 mm d^-1^ (8-day interval, cp. Figure 4). In general, the spatial differences of ET in the grasslands of the Xilin river catchment were highest during periods of high mean ET, as from DOY 185–192 in 2003, when ET varied between 0.7 mm d^-1^ and 5.2 mm d^-1^. However, for the most part of the data (90%), ET was between 1.5 and 3.5 mm d^-1^ at that time, indicating exceptional dry or wet local conditions or a misclassification of a few pixels led to these extreme differences.

Despite all the identified variations of ET, the results showed a primary spatial pattern of long-term ET (10 years). The pattern can be explained partly by the effects of longitude and altitude, since ET generally decreases in western directions (R^2^ = 0.35) and in lower altitudes (R^2^ = 0.38). The fundamental relationship of both factors and long-term ET, is even more pronounced when they are combined (R^2^ = 0.49). On the contrary, the influence of latitude on ET is little and can be neglected (R^2^ = 0.07).

In the study of Schaffrath et al. (2013) ET approximately balanced P in the study area in 2006 and this study confirms this in general in the long-term (ET/P = 0.99, Table 2). The ET-studies of Miao et al. (2009), Lu et al. (2011) and Wang et al. (2012) also report ET to approach or exceed P in this area. Excess ET over P during the vegetation period, which was also modelled in our study from 2004 on, is related to a decrease of soil water storage during this period. However, the linear character of the annual relationship between P and ET was not applicable in 2003, when P was clearly above average (301 mm) and ET was 199 mm only. Some of this moisture was stored in the soil (25 mm) but the bulk (77 mm) was modelled as vertical gravitational flow through the parameterised soil profile indicating a possible groundwater recharge in this exceptionally wet year. However, due to the diverse geology in the basin with deep layers of Quaternary deposits and volcanic rocks (Barthold et al. 2013) and the simplified parameterisation of a soil profile with 1.2 m depth only, the water flow pathways are a bit speculative. But the geological setting and the existence of groundwater-irrigated agricultural land in the basin indicate the existence of groundwater aquifers, which may be recharged during an exceptional wet year like in 2003. However, the enormous difference between P and ET in 2003 may not be regarded to be a common phenomenon in high P years, because a significant amount of P in 2003 occurred during a short period (Figure 4). A more balanced distribution of high annual P over a longer period and many smaller P-events will most likely increase ET, while the water flow into deeper layers is reduced. The study of Lu et al. (2011), which analysed ET-soil water relationships in the grasslands of Inner Mongolia, also showed the larger the rainfall events, the deeper the pulses in soil moisture change. Also, in their study, small events below 6 mm did not even cause any sustained changes of soil moisture in the upper soil layers.

A strength of this study and an advantage over the MOD16 ET data is the transfer of the matrix potential (ψ, every 10 cm) of every pixel and time-step and from 31 December to 1 January of the following year, because it allowed for the effects of inner- and interannual soil water storage variations. This could explain the finer increments of ET and the higher values of spring ET of our results. The variations of ψ (as simulated by BROOK90) were more pronounced in the upper soil layers: expressed as the mean value of the 3029 pixels of the study area, ψ of 31 December varied between −23 kPA in a very wet year like 2003 and −90 kPA in a dry year like 2009 at the 0.1 m level (σ = 19 kPA, n = 10), whereas ψ at a depth of 1.1 m just varied between −17 kPA in 2003 and −51 kPa in 2009 (σ = 10 kPA, n = 10).

The low spring ET values and high contrast between adjacent pixel of the spatial 8-day ET are limiting the MODIS MOD16 ET data for local ET studies in this area. Nevertheless, the good agreement of the spatial patterns , 8-day ET mean values (R^2^ = 0.63) and the long-term (10 years) spatial pattern of ET between both data sets (R^2^ = 0.79) indicate the reasonableness of our modelled ET and the potential of the MODIS MOD16 ET data for long-term grassland studies. The MOD16 ET data is available from 2000 to 2012, however, according to the project description, it will not be processed in near-real time (http://ntsg.umt.edu/project/mod16, accessed 19 June 2013).

Schaffrath et al. 2011 showed the spatial and temporal variability of LAI is controlled by the temporal and spatial distribution of P events during the growing season and this study showed the variability of ET to be dependent on P as well. The multidimensional view on ET in this study (temporally from 8-day, and yearly up to a ten-year period and spatially from 1 km^2^ to the complete typical steppe land use unit of the Xilin river catchment) allowed for the quantification of the variability and the identification of individual ET (8-day, yearly) and a general (10 years) map of the long term ET over the last decade. Both, the high innerannual and the interannual variation of ET and its variable pattern stress the need for the adaptation of static land use regimes towards dynamic land use strategies and the remarkable position of traditional nomadic pastoralism in this area. Nomadism is regarded as a traditionally adapted socio-economic way of culture which is adjusted to the variable carrying capacity of the environment. This traditionally common lifestyle in drylands worldwide proved to be sustainable over at least two thousand years by grazing according to the environmental conditions (Scholz 1995). Apart from the important economical value and function of the grasslands today, they also provide a natural and ecological barrier against the vast deserts of Central Asia for Central China as grassland soils and surfaces show: particles deflated in deserts are accumulated in these grasslands under natural conditions (Reiche et al. 2012b). The recent and ongoing land use change showed the current livestock management to be poorly adapted resulting in the degradation of these fragile ecosystems (Zhu and Wang 1993 Zhou et al. 2002 Chen et al. 2003 Tong et al. 2004 Jiang et al. 2006 Han et al. 2009). We consider this spatial study to be a highly useful motivation for the restoration of the grasslands through development of a sustainable rangeland management, which by adjusting numbers and distribution of livestock according to the conditions takes into account the inherent variations of P, LAI and ET in this area.

### Uncertainties and potential sources of error

The accuracy of our ET estimations is highly dependent on the quality and accuracy of the used input data. One important potential error source is P. Several issues exist: P may be under estimated occasionally, because it is not always followed by a gain in LAI and thus can not be detected (Schaffrath et al. 2013). Another inherent issue of estimating P from LAI gain is the gap between a possible P event and the gain of grassland LAI. The 8-day intervals of MODIS LAI and our estimated P will smooth this effect but can also lead to an inconstant temporal bias in P estimations of individual periods. We are also aware of the possible uncertainty of the statistically scaled 1-day P on 1-day ET model results and thus provide the results from the 8-day resolution on. Another potential error source of P is introduced by using the mean relationship between P and LAI from data of all of the six stations, because they vary site specifically. However, the extended model used for this study strengthens the robustness of the relationship between MODIS LAI gain and measured P (R^2^ = 0.80, n = 270 instead of R^2^ = 0.76, n = 0.76). This is in agreement with the mean percentage error of modelled P, which was 21% only when compared to the measured P at the six stations.

Some other generalisations of input data may influence the results as well: minimum and maximum air temperatures are mean values of eight-day periods and thus day-to-day variations are neglected. This is certainly a higher uncertainty than the error introduced by the calculation of Ta_min and Ta_max from MODIS Ts. Also, input data between 29 August and 22 April does not result from remote sensing data because our method for P estimation does not work without vegetation growth and uncertainties and data gaps of MODIS Ts due to snow cover may not allow for the estimation of Ta during this period. Thus, micrometeorological data of IMGERS and EC-stations of the MAGIM project were used for all of the 3029 pixels and spatial characteristics could not be taken into account. This is a simplification but the impact of this generalisation on modelled ET is relatively small because most of the P (70%) occurs between May and August (Chen et al. 2005), ET is low in winter and the spatial variability of ET is still low in spring.

There are also several issues regarding the use of MODIS LAI: MODIS LAI tends to be higher than the actual LAI in this area. This could result in the overestimation of modelled ET under wet conditions. However, our modelled ET shows reasonable values, which are in the range of published field data in this area, e.g., Hao et al. (2007,2008) and Wang et al. (2012). We also observed an increase of MODIS LAI after the shift from collection 4 to collection 5 of the data product. The actual course of LAI over the year is implemented in BROOK90 by 10 possibilities to adjust LAI changes and date, which is a slight limitation in the study area because some smaller changes in LAI are neglected. Canopy height is represented as a function of LAI, which is a rough estimation, too. There are also only ten dates available to set canopy height in BROOK90.

Modelled ET is also based on one representative soil profile only, which is a generalisation of the distribution of soils in the study area. However, the analysis of available soil data throughout the area showed some variation in horizon depths and texture, but always sand to be the dominating particle fraction. We examined the impact of 18 different soil profiles on modelled ET of a 4x4 pixels area (cp. Vetter et al. 2012) and modelled ET for the whole study area with the three soil profiles which resulted in the lowest, mean and maximum ET (Schaffrath et al. 2013). The influence of soil texture on ET was found to be highest in spring (±25%) and significantly lower (±10%) in summer when ET is dominated by T. In general, the results of modelled ET varied between −11% and +6% from ET modelled with the soil profile used in this study. The application of a soil map as modelled by Barthold et al. (2013) may reduces the uncertainty; however, the map does provide the mean soil texture for the Ah horizon of soil groups according to the WRB (IUSS Working GROUP WRB 2007) only, without detailed information of deeper soil layers. The use of model generated solar radiation and a constant wind speed does not affect the general trends of ET; however, small additional systematic effects of their daily variations on ET may be undetected (Vetter et al. 2012). Some features of the model BROOK90 could impact the accuracy of modelled ET as well: there is no provision for dew, rime and variations of albedo; also, the model does not include non-green leaves which may intercept solar radiation but do not transpire.

All the uncertainties from the daily input data and the generalisation of parameters could introduce errors or biases in modelled ET, which are difficult to detect and quantify.

## Conclusions

The study clearly shows the spatial variability and temporal dynamics of ET in this water-limited region over a ten-year period. The study also emphasises the importance of long-term investigations in semi-arid grassland ecosystems, where atmosphere-plant and soil interactions are highly variable, since a wide spectrum of variations of natural conditions in both, time and space, may not be detected in a short study period of a few months or years. This paper also revealed the importance of spatial ET results, which are superior to point scale measurements, by, e.g., the EC-technique, and highly useful to enhance the understanding of ecosystem functioning particularly in sparsely populated and semi-arid regions. Despite some limitations due to the 8-day temporal and 1 km spatial resolution of the method applied, we found a long-term spatial pattern of ET, which is partly based on longitude and altitude and a high spatial, innerannual and interannual variability of ET, which is triggered by the distribution of larger P-events. The high variability of ET indicates the advantage of dynamic land management practices over static land use regimes in Inner Mongolian grasslands.

Therefore, based on these findings and in order to protect and restore the grassland ecosystems of Inner Mongolia, the need for the adaptation of static land use regimes towards intelligent and dynamic land use strategies, which control grazing and stocking rates according to the variable environmental conditions and the remarkable position of the sustainable practice of nomadic pastoralism in this area are stressed.

## Abbreviations

CV: Coefficient of variation
DOY: Day of the year
E: Soil evaporation
Ei: Evaporation from intercepted rain
EC: Eddy-covariance
ET: Evapotranspiration
gc: Canopy conductance
IMGERS: Inner Mongolia grassland ecosystem research station
LAI: Leaf area index
MAGIM: Matter fluxes in grassland ecosystems of Inner Mongolia
MODIS: Moderate resolution imaging sprectrradiometer
PM: Penman-Monteith
P: Precipitation
PDF: Probability density function
SD: Standard deviation
SRTM: Shuttle radar topography mission
T: Transpiration
Ta_min: Minimum air temperature
Ta_max: Maximum air temperature
Ts: Surface temperature
VPD: Vapour pressure deficit
WRB: World reference base for soil resources.
